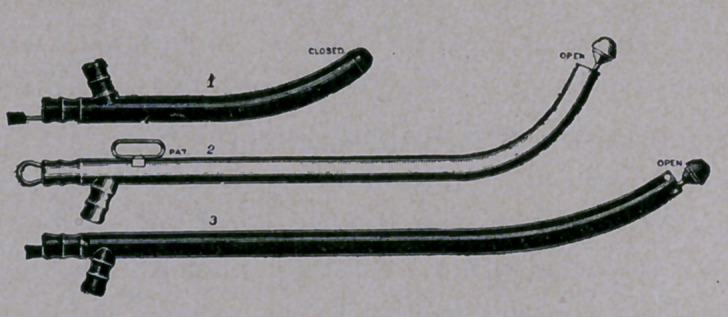# Catheters and Cystitis

**Published:** 1899-02

**Authors:** R. N. Mayfield

**Affiliations:** New York


### Catheters and Cystitis.

BY R. N. MAYFIELD, M. D., NEW YORK.

It is well known that when it is necessary to use a catheter of usual construction—that is, with the ordinary fine perforations as an inlet thereunto—it does not work readily or satisfactorily, or subserve fully the results expected from it.

Examples of such unsatisfactory operations are seen where there is a good deal of mucus present in the bladder, such mucus being apt to surround or lie upon the end of the catheter, clogging or stopping the apertures thereof and preventing the ingress of fluids to be drawn off; again, when sediment or calcareous matter is present, it clogs, even sometimes filling in part or completely the apertures, with consequent failure of the catheter to fully perform its functions. Such failures are especially apt to happen in nearly, if not quite, all forms of chronic diseases of the bladder, and notably so in cystitis.

My object, therefore, is to present a catheter that is reliable and efficient in operation when the use of a catheter is indicated in all conditions and diseases of the bladder. In this instrument the danger of clogging or failure to perform its functions is obviated, and its interior may be readily made aseptic, and bits of mucus that usually clog an ordinary catheter may be readily drawn off.

This catheter is of very simple construction, being tubular, with the curve of an ordinary instrument, and opened at the end for an inlet. For the closure of this open end, and for the easy insertion of the catheter, as well as for other purposes, a bulbous or rounded head is used, preferably solid, and attached to one end of a wire, passing through the body or tube and projecting at its rear or outlet end.

This construction forms a very efficient catheter having an area of opening so large as to greatly obviate the danger of clogging, for, if mucous should lodge against the open end, the working of the head back and forth upon its seat would cut away the obstructing bits of mucus and permit them to pass through the tube.

With this instrument there should be no hesitancy in using nitrate of silver, iodine, corrosive sublimate, carbolic acid, or hydrogen solutions, in the bladder, as any of these solutions can be readily drawn off or neutralized, thus preventing poisoning from absorption, or preventing ruptures from gases that form in the bladder.

Regarding the treatment of cystitis with the employment of this catheter, presuming that we have a typical case, with ropy, viscid, and tenacious mucus, the membrane thickened and possibly ulcerated, and in deep folds—"ribbed," as it were—we begin the treatment as follows:

1. Inject a quarter of a grain of cocaine dissolved in a drachm of water into the membranous portion of the urethra.

2. Anoint the largest hard-rubber catheter that can be well passed into the bladder, and increase the size one number each week until the urethra is normal size.

3. Begin with dilute hydrogen solutions—preferably hydrozone—one part to twenty of lukewarm water, using this solution freely, especially when employing the large size catheter. If the small size is used at the beginning, I recommend the use of only two or three ounces at a time until removed by the return flow. This can be repeated until the return flow is clear and not "foaming," which indicates that the bladder is aseptic.

4. Partly fill the bladder with the following solution: tincture of iodine compound, two drachms; chlorate of potassium, half a drachm; chloride of sodium, two drachms; warm water, eight ounces. Let it remain a minute or so and then remove. This treatment should be used once or twice a day.

Where I suspect extensive ulceration I recommend once a week the use of from ten to twenty grains of nitrate of silver to the ounce, and neutralize with chloride-of-sodium solutions.

This treatment carried out carefully will be satisfactory, as there is no remedy that will destroy bacteria, fœtid mucus, or sacculated calcareous deposits like hydrozone.

## Figures and Tables

**Figure f1:**